# Isolation an Antimicrobial Susceptibility Profile of *Edwardsiella* Species and *Escherichia coli* O157:H7 From Fish and Their Handling Environment in Lake Abaya and Gilgel Gibe‐3 Dam, Southern Ethiopia

**DOI:** 10.1155/vmi/4857001

**Published:** 2026-10-07

**Authors:** Belay Walelign Moges, Jelalu Kemal Birmeka, Bruk Abraha Fitwi, Bulako Chebo Boke, Tesfahun Demeke Dana, Tesfaye Bekele Kassa

**Affiliations:** ^1^ Department of Veterinary Science College of Veterinary Medicine and Animal Science Jinka University Jinka Ethiopia; ^2^ College of Veterinary Medicine Haramaya University Haramaya Ethiopia haramaya.edu.et; ^3^ School of Veterinary Medicine Wolaita Sodo University Wolaita Sodo Ethiopia wsu.edu.et

**Keywords:** antimicrobial resistance, *Edwardsiella* species, *Escherichia coli*, fish, isolation

## Abstract

*Edwardsiella* species are reassigned under the family Hafniaceae following phylogenomic revisions of the order *Enterobacterales*, a primary pathogenic strain of fish, while *Escherichia coli* are members of the *Enterobacteriaceae* family that are frequently reported as contaminants with rare causes of illness in fish but severely affect other mammals. A cross‐sectional study was conducted from April to November 2022 with the objectives of estimating the prevalence of *Edwardsiella tarda*, *Edwardsiella ictaluri*, and *E. coli* O157:H7 in fish and determining the antimicrobial susceptibility pattern of the isolates. A total of 768 tissue samples comprising intestine, liver, and kidney were collected from 256 apparently healthy fish. Bacterial isolation was performed using selective and differential culture media, including MacConkey agar, xylose lysine deoxycholate (XLD) agar, *Edwardsiella* isolation medium, eosin methylene blue (EMB) agar, and sorbitol MacConkey agar. Further identification was performed using biochemical tests and latex agglutination assays, particularly for the targeted strain of *E. coli*. The disk diffusion test was employed to determine the susceptibility pattern of isolates against seven standard antimicrobial disks. The findings were presented using descriptive statistics, while the association of categorical variables with the occurrence of bacterial species was computed using chi‐square and logistic regression analysis. The overall prevalence of *E. tarda, E. ictaluri,* and *E. coli* O157:H7 among fish was 8.6%, 4.7%, and 14.1%, respectively, and among organs (intestine, liver, and kidney) was 4.01%, 1.95%, and 5.34%, respectively. The maturity of fish and lake showed a significant association (*p* < 0.05) with the occurrence of *E. tarda*. Resistance to tetracycline was noted in all of the isolates. The results of this study concluded that the intestines of fish harbor *E. tarda, E. ictaluri,* as primary inhabitant pathogens, and *E. coli* O157:H7, which may be a transient microorganism mostly associated with fault handling practice.

## 1. Introduction

Fish farming is a rapidly growing sector in various parts of the world. This is due to the fact that products of fish are valuable sources of food and are economically important[1–3]. Fisheries play a substantial role in food security. In addition, it serves as a source of income, improvement in livelihood, and social development in developing countries [4]. Dramatic growth in aquaculture production in the past two decades has promoted the average consumption of fish and fishery products globally [5]. Whole fish currently make up about 19% of the total protein consumption [6] Ethiopia depends on inland water, including about 7400 km^2^ of lakes and reservoirs and about 7000 km of rivers with an estimate of more than 200 edible fish species [1, 7]. In Ethiopia, the highly increasing fish demand is mainly met through inland capture fishery and aquaculture reservoirs [2]. About 75% of the total fish catch originates from main water bodies, such as Ziway, Hawasa, Tana, Langano, Chamo, and Abaya, and the remaining 25% originates from minor lakes, reservoirs, and rivers [8]. Ethiopia has an endowed diversity of ichthyofauna in its lakes, rivers, and reservoirs [9].

*Escherichia coli* (*E. coli*) are a Gram‐negative, rod‐shaped, nonacid‐fast, uniform‐staining bacterium that belongs to the *Enterobacteriacea*e family*. E. coli* is a common conditional pathogenic bacterium in the intestines and environment of humans and animals [10]. *E. coli* also causes diseases in different aquatic animals and results in serious economic losses for the aquaculture industry. Most of the bacteria that are normal inhabitants in the small intestine and colon are nonpathogenic. Nevertheless, this nonpathogenic *E. coli* causes disease if it spreads outside of the intestine to other organs [11].

The pathogenic serotype of *E. coli* O157:H7 may cause diarrhea by producing and releasing toxins (called enterotoxigenic *E. coli* or ETEC) and may be the cause of food spoilage in fish [12]. *E. coli* O157: H7 is one of the most important serotypes that contains pathotypes that cause food‐borne infection in humans, animals, and fish, and it has been found in the intestines of healthy animals and fish [13].

Several species historically placed in *Edwardsiella* (family *Enterobacteriaceae*) have been reassigned under the family Hafniaceae following phylogenomic revisions of the order *Enterobacterales*. From the genus *Edwardsiella*, fish are usually infected by either *Edwardsiella tarda* (*E. tarda*) or *Edwardsiella ictaluri (E. ictaluri)*, whereas *Edwardsiella hoshinae* usually infects reptiles and birds [14]. *Edwardsiella* species are known pathogens affecting the fish population worldwide [15–17].

*E. tarda* is among the most important bacterial diseases causing severe economic losses, reduced growth, and increased cost of production [18]. It leads to devastating fish disease prevailing worldwide [19, 20]. *E. tarda* is pathogenic to wide range of fishes, such as Nile tilapia (*Oreochromis niloticus*), catfish (*Clarias gariepinus*), Nile perch (*Lates Niloticus*), Silver catfish (*Bagrus docmak*), Channel catfish (*Ictalurus punctatus*), carp (*Cyprinus cyrpio*), and Flounder (*Paralichthys olivaceus*) [21].

*E. tarda* causes a disease commonly known as fish gangrene which is an emphysematous putrefactive disease. *E. tarda* infections in fishes are usually associated with high temperatures, overcrowding, poor water quality, and the presence of high concentrations of organic matter [20].

*E. ictaluri* is recognized as a disease of catfish species and is known to be an economically important bacterial disease that can survive in both freshwater and marine ecosystems [17]. The organism is the most widespread species from the genus *Edwardsiella*, which has been detected in over 20 species of freshwater and marine fish from 25 countries in America, Asia, Europe, Australia, the Middle East, and Africa [5, 22].

Fish disease control is particularly difficult because fish are often farmed in a system where production is dependent on natural environmental conditions [19]. To overcome the problem, multidisciplinary approaches involving the characteristics of potentially pathogenic microorganisms for fish, aspects of the biology of fish, and a better understanding of the environmental factors affecting such cultures allow the application of adequate measures to prevent and control the diseases limiting fish production [23].

Indiscriminate use of drugs to treat bacterial diseases in fish promotes the development of drug‐resistant bacteria [24]. It is challenging to treat drug‐resistant bacteria with common antibiotics. Different bacteria, such as *E. tarda, E. coli,* and antimicrobials, are released into water bodies through the excretion of animals and human feces. These phenomena result in the infection of aquatic animals with different bacteria and the development of drug‐resistant bacteria [25].

Studies conducted by Ref. [26–28] showed the occurrence of *E. ictaluri, E. tarda*, and *E. coli* O157:H7, such as 1.2%, 19.05%, and 4%, respectively, in freshwater fish of Ethiopia. However, the importance of *E. tarda*, *E. ictaluri*, and *E. coli* O157:H7 in fish disease has not been well documented. In particular, there is limited information on the selected study areas of Lake Abaya and Gilgel Gibe‐3 Dam on the isolation of *Edwardsiella* species (*E. tarda* and *E. ictaluri*) and *E. coli* O157:H7. Knowledge and understanding on the status and antimicrobial resistance profile of *E. tarda*, *E. ictaluri,* and *E. coli* O157:H7 in areas where there is high fish production potential is important in the design of protocols to tackle the existing disease burden. Therefore, this study was undertaken at Lake Abaya and Gilgel Gibe‐3 Dam with an overall objective of investigating the epidemiology of *E. tarda*, *E. ictaluri*, and *E. coli* O157:H7 in different fish species.

## 2. Materials and Methods

### 2.1. Description of the Study Areas

The study was conducted at Lake Abaya and Gilgel Gibe‐3 Dam. Lake Abaya is the largest of the Ethiopian Rift Valley Lakes and is located north of Lake Chamo and south of Abala Abaya Woreda and 375 km far to the south of Addis Ababa, the capital city of Ethiopia. This lake is found at an altitude of 1165.459 m above sea level with geographic coordinates of 6°35′55″NL and 37°53′0″EL, and it occupies an area of 1070 km^2^ with a maximum depth of 13.1 m and an average depth of 7.1 m. The maximum and minimum daily temperatures are 35°C and 20°C, respectively [29].

The Gilgel Gibe‐3 Dam is located about 420 km of the southwest of Addis Ababa at 6°54′ 12″NL and 37°26′46″EL and at an altitude of 902.694 m above sea level. Administratively, the reservoir bordered five zones, 11 districts, and 67 *kebeles*. Gilgel Gibe‐3 Dam is an area under the jurisdiction of the Loma district of the Dawro Zone and the Kindo Didaye and Kindo Koysha districts of the Wolaita Zone of the Southern Nations, Nationalities, and People Regional State [30]. The total area is estimated to be 17,187 ha of land. The rain pattern is a bimodal distribution, with a short rainy season extending from January to April and long rains from June to September. The average annual rainfall is 904 mm. The minimum and maximum daily temperatures are 21°C and 29.20°C, respectively [31]. The map of the study area is presented below in Figure 1.

**FIGURE 1:**
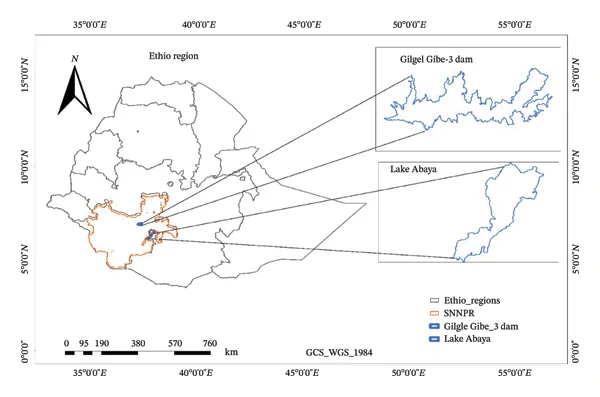
Map of study area (Lake Abaya and Gilgel Gibe‐3 Dam) (*Source:* [32]).

### 2.2. Study Population

The study was carried out on apparently healthy fish species that were caught by local fisherman and harvested for human consumption including Nile tilapia (*Oreochromis niloticus*), catfish (*Clarias gariepinus*), and Nile perch *(Lates niloticus)* from Lake Abaya, whereas Nile tilapia (*Oreochromis niloticus*), catfish (*Clarias gariepinus*), and Belchi fish (*Vericorhinus beso)* were from Gilgel Gibe‐3 Dam. All fish, regardless of their sex and age, were considered for the study. The age of fish was determined by counting the annuli (individual rings), spacing between rings, hard spine, and hard structure of the fish, and categorized as immature (I–II years) and mature (III–V years) as described by Ref. [28].

### 2.3. Study Design

A cross‐sectional study was undertaken to estimate the prevalence of *E. tarda*, *E. ictaluri*, and *E. coli* O157:H7 and to determine their antibiotic susceptibility from April to November 2022 on apparently healthy fish species harvested for human consumption from Lake Abaya and Gilgel Gibe‐3 Dam.

### 2.4. Sampling and Sample Size Determination

The four fish species were deliberately chosen based on their popularity among local fishermen and consumer demand in the targeted water bodies. The sample size required for this study was determined depending on an expected prevalence of *E. tarda*, *E. ictaluri*, and *E. coli* O157:H7 with a 95% confidence interval and 5% absolute precision according to [33] as follows:

(1) N=1.962XPexp 1−pexpd2,

where *N* = required sample size, *P*_exp_ = expected prevalence of the organisms in fishes, and *d* = desired absolute precision of 5%.

Based on the previous study’s prevalence of 20.59% [18], the calculated sample size was 256 fish. Thus, a total of 170 fish from Lake Abaya (68 Nile tilapia, 68 Nile perch, and 34 catfish) and 86 fish from Gilgel Gibe‐3 Dam (33 Belchi fish, 33 Nile tilapia, and 20 catfish) were sampled randomly. The proportion is taken based on the overall fish catch composition of the fish species as described in previous studies [34, 35]. The final sampling comprises three separate samples from each fish, which include the intestine, liver, and kidney. Thus, the overall number of samples subjected to laboratory analysis is 768.

### 2.5. Sampling Procedure and Data Collection

Fish were grouped into identical species, and then, individual fish species were randomly sampled depending on the number of fish species caught on each day. Samples were collected weekly. On each visit, 12 fish tissue samples (intestine, liver, and kidney) were collected. During collection, each fish was legibly and clearly labeled with identified information, such as date of sample collection, fish species, sex, age (maturity), and lake (source) of fish. The samples were then packed separately in a sterilized plastic bag and immediately transported using an ice pack to the Wolaita Sodo University, School of Veterinary Medicine Microbiology Laboratory. Upon arrival, each fish was surface decontaminated by 70% alcohol and incised along the ventral midline of the abdomen, starting from the anus up to the mouth using a sterile dissecting scalpel blade, followed by another dissection from the anus to the lateral line and farther along the lateral line up to the gill cover to remove the lateral side of the abdominal wall so as to expose the internal organ. The required tissue samples were carefully removed from the organs and placed in separate sterile test tubes containing 10 mL of buffered peptone water (BPW) and shaken by a high‐speed vortex mixer for 30 s so as to allow uniform distribution of microorganisms. Then, the samples were incubated at 37°C for 24 h for *E. tarda* and *E. coli,* whereas at 30°C for 24 h for *E. ictaluri* according to the methods described by Ref. [36, 37].

### 2.6. Isolation and Identification of *Edwardsiella* Species

The bacteriological media used in different stages of the isolation of *Edwardsiella* were prepared according to the manufacturer’s recommendations. A loop full of culture from the BPW enrichments was streaked on a MacConkey agar plate and incubated for 24 h at 25°C for both *E. ictaluri* and *E. tarda* [38]. A loop full of inoculums from MacConkey agar plate, a loopful colony, was streaked onto xylose lysine deoxycholate (XLD) agar and *Edwardsiella* isolation medium (EIM) agar plates and incubated at 25°C for 48 h. Then, the plates were examined for the presence of typical Edwardsiella colonies, in that *Edwardsiella* species on XLD agar appear as small, circular, raised, whitish colonies with black centers due to H_2_S production. On EIM agar, *E. tarda* and *E. ictaluri* appear as colorless with black centers and green colonies, respectively.

For identification, at least five presumptive *E. tarda* and *E. ictaluri* colonies were selected from each medium plate and streaked onto nutrient agar plates in a manner that allowed getting isolated colonies. The inoculated media were incubated for 24 h at 25°C for both E. *tarda* and *E. ictaluri,* respectively. For categorization of the isolates to species level, gram stain, catalase, motility, oxidase, sugar fermentation test (i.e., glucose, lactose, and sucrose fermentations), H_2_S production (i.e., in TSI agar), MR, VP, citrate, and indole production tests were employed [39].

### 2.7. Isolation and Identification of *E. coli* O157:H7

*E. coli* was isolated and identified according to the methods outlined in the International Organization for Standardization [40, 41]. The media used for isolation and identification of *E. coli* were prepared according to the manufacturer’s recommendations. All samples were processed separately, and then, the processed samples in appropriate amounts, 10 mL of BPW, were incubated at 35°C for 24 h. A loop full of culture from the BPW enrichments was streaked on MacConkey agar plate and then incubated at 35°C for 24 h. The typical colony on MacConkey agar with pink color was transferred to eosin methylene blue (EMB) agar. The colonies with metallic sheen on EMB agar were transferred to nutrient agar for further primary identification (gram staining, catalase, oxidase, and OF tests) as well as confirmatory biochemical tests using the IMViC test. *E. coli* isolates were transferred to sorbitol MacConkey agar to detect the presence of *E. coli* O157:H7, which is indicated by the presence of colorless or straw‐colored colonies [42]. Final confirmation of *E. coli* O157:H7 was done based on the latex agglutination test.

To perform the test, 40 μL drops were placed in two circles on the reaction cards for each sample. For each test sample, one drop of the O157:H7 test latex was added to one circle, and one drop of the O157:H7 test latex was added to the other circle. The contents of the samples were mixed and spread over the entire circle area. Samples were inspected for the presence of agglutination after 1 min [43].

### 2.8. Antimicrobial Susceptibility Test

Antimicrobial susceptibility was done with the disk diffusion method using Mueller–Hinton agar. First, a suspension of a sample in a saline solution was prepared by adjusting it with the 0.5 McFarland turbidity standards. Pure culture identified as *E. tarda, E. ictaluri*, and *E. coli* O157:H7 colonies was taken from nutrient agar and transferred into a test tube containing 5 mL of a sterile saline solution and mixed gently to make a homogenous suspension. A cotton swab was used to strike the bacteria across the surface of Muller–Hinton agar and wait for about 3 min to dry [44].

Antibiotic disks were placed on the cultured agar surface using clean and sterile forceps. The antimicrobial agents used in this study were streptomycin (10 μg), cefoxitin (30 μg), ampicillin (10 μg), tetracycline (30 μg), gentamicin (10 μg), sulfamethoxazole (100 μg), and trimethoprim (5 μg). The source of all antibiotic disks obtained from the Thermo Fisher Scientific (Oxoid) supplier. Following this, plates were aerobically incubated at 25°C for 24 h for *E. tarda, E. ictaluri isolate,* and *E. coli* O157: H7 isolates at 35°C for 20 h. Finally, the diameters of the zone of inhibition around the disks were measured to the nearest mm using a digital electronic caliper, and the isolates were classified as susceptible, intermediate, and resistant to the drugs tested according to the interpretation standards of the Clinical Laboratory Standards Institute [44, 45] as presented below (Table 1).

**TABLE 1:** Antibiotic disks used for susceptibility test against the targeted organisms and their respective concentrations.

Antibiotic disk	Disk	Zone of inhibition to the nearest mm		
	Concentration (μg)	Susceptible (≥)	Intermediate	Resistance (≤)
Streptomycin	10	15	12–14	11
Cefoxitin	30	18	15–17	14
Ampicillin	10	17	14–16	13
Tetracycline	30	15	12–14	11
Gentamycin	10	15	13–14	12
Sulfamethoxazole	100	16	11–15	10
Trimethoprim	5	16	11–15	10

*Note:* Source: [45].

### 2.9. Data Management and Analysis

All the recorded and coded data were entered into a Microsoft Excel spreadsheet (Microsoft Corporation) and checked for accuracy. After validation, the data were transferred to IBM SPSS Software Version 26 (IBM, Chicago, USA) for analysis. The prevalence of bacterial species was defined as the number of *E. tarda*, *E. ictaluri*, and *E. coli* O157:H7‐positive samples per the number of samples examined. The fish were considered positive when the examined tissue samples (intestine, liver, and kidney) were culture‐positive for *E. tarda*, *E. ictaluri*, and *E. coli* O157:H7.

The chi‐square and multivariable logistic regression analyses were employed to establish the association between cultured bacteria result and risk factors. In addition, Fisher’s exact test with two‐tailed test analyses was considered for variable categories that did not fulfill the minimum expected counts. Before multivariable logistic regression analysis, the data were checked for fulfillments of assumptions, such as correlation of each variable (not more than 0.7), multicollinearity tests (tolerance > 0.1), and the association of independent variables with the dependent variable (*p* ≤ of 0.2). Thus, lake and fish types were not considered in the final model for *E. coli* O157:H7 analysis. The odds ratio (OR) was computed at 95% CI, and in all cases, the difference between parameters was considered significant at *p* < 0.05.

## 3. Results

### 3.1. Fish Composition in the Studied Lakes

Among the fish samples caught for microbiological examination, the majority of the fish were *L. niloticus* (101, 39.5%) followed by *O. niloticus* (68, 26.6%), *C. gariepinus* (54, 21.1%), and *V. beso* (33, 12.9%). The majority of the fish taken in this study were from Abaya Lake 170 (66.4%), while the number of fish from Gilgel Gibe‐3 Dam was 86 (33.6%). Regarding the sex of fish, 147 (57.4%) were males and 109 (42.6%) were females. The maturity of the fish comprises about 75 (29.3%) immature and 181 (70.7%) mature (Table 2).

**TABLE 2:** The prevalence of E. tarda, E. ictaluri and E. coli O157:H7 with considered variables.

Variables	Categories	Frequency	Percent (%)
Sex	Male	147	57.4
	Female	109	42.6

Site	Lake Abaya	170	66.4
	Gibe‐3 Dam	86	33.6

Maturity	Mature	181	70.7
	Immature	75	29.3

Fish spp.	*O. niloticus*	68	26.6
	*L. niloticus*	101	39.5
	*C. gariepinus*	54	21.1
	*V. beso*	33	12.9

	Total	256	100

### 3.2. Prevalence and Associated Risk Factors

Of the total of 256 fishes examined for the presence of bacteria, 36 (14.1%), 22 (8.6%), and 12 (4.7%) were positive for *E. coli* O157:H7, *E. tarda,* and *E. ictaluri,* respectively. The proportion of *E. coli* O157:H7 tends to be higher in female (18.3%) than male (10.9%) fishes. However, no significant difference (*p* > 0.05) was observed in the proportion of the isolates among sex categories. The age of fishes showed significant association (*p* < 0.05) with the occurrence of bacterial species; in that, it was higher in immature fishes than mature ones. Among the bacterial species, *E. tarda* showed significant association (*p* < 0.05) with the source fish, in that a higher proportion was found in Gilgel Gibe‐3 Dam (14%) than Abaya (5.9%) lakes (Table 3). Among the fish species examined, *C. gariepinus* was the only species to harbor *E. ictaluri* at a proportion of 22.2%, whereas it was the species to have a low proportion of *E. coli* (9.3%) and *E. tarda* (1.9%) isolates as compared to the other fish species (Table 3); the following (Figures 2, 3, and 4) are some pictures picked during laboratory examinations for isolations of *E. coli* O157:H7 in particular.

**TABLE 3:** Risk factors associated with the occurrence of bacterial species isolated from freshwater fish of Lake Abaya and Gilgel Gibe‐3 Dam.

Variables	Categories	No. of fish examined	No. (%) of fish positive to:		
			*E. coli* O157:H7	*E. tarda*	*E. ictaluri*
Sex	Male	147	16 (10.9)	10 (6.8)	9 (6.1)
	Female	109	20 (18.3)	12 (11)	3 (2.8)
	Total	256	36 (14.1)	22 (8.6)	12 (4.7)
		*χ* ^2^ (*p* value)	2.9 (0.09)	1.4 (0.235)	1.6 (0.207)

Age	Mature	181	20 (11)	11 (6.1)	5 (2.8)
	Immature	75	16 (21.3)	11 (14.7)	7 (9.3)
	Total	256	36 (14.1)	22 (8.6)	12 (4.7)
		*χ* ^2^ (*p* value)	4.6 (0.031^∗^)	4.9 (0.02^∗^)	5.1 (0.04^∗^)

Lake	Abaya	170	24 (14.1)	10 (5.9)	6 (3.5)
	Gilgel Gibe‐3 Dam	86	12 (14)	12 (14)	6 (7.0)
	Total	256	36 (14.1)	22 (8.6)	12 (4.7)
		*χ* ^2^ (*p* value)	0.001 (0.972)	4.7 (0.03^∗^)	1.5 (0.226)

Fish type	*O. niloticus*	68	11 (16.2)	8 (11.8)	0
	*C. gariepinus*	54	5 (9.3)	1 (1.9)	12 (22.2)
	*L. niloticus*	101	15 (14.9)	7 (6.9)	0
	*V. beso*	33	5 (15.2)	6 (18.2)	0

	Total	256	36 (14.1)	22 (8.6)	12 (4.7)
		*χ* ^2^ (*p* value)	1.7 (0.713)	8.2 (0.04^∗^)	47.1 (0.001^∗^)

*Note:* ^∗^The variable/category showed statistically significant association.

**FIGURE 2:**
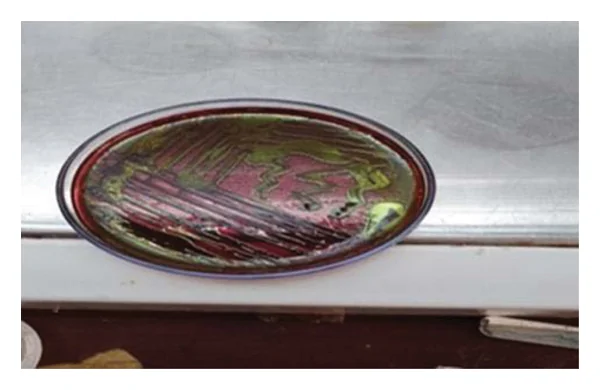
Presumptive *E. coli* colony on EMB agar done in the Haramaya University College of Veterinary Medicine Microbiological Laboratory.

**FIGURE 3:**
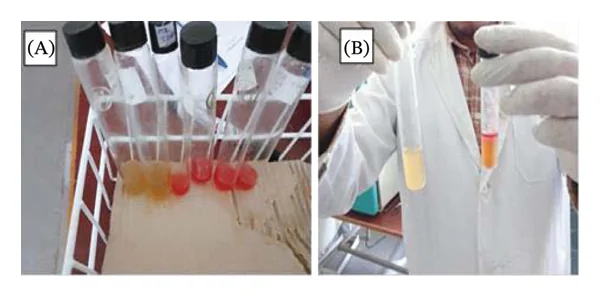
Methyl Red and Indole Test. (A) The first four right tubes indicate a red color that shows MR‐positive, and the two left‐sided tubes remain yellow, indicating that MR‐negative; (B) Indole test, the right pinkish‐ringed tube indicates indole‐positive, while the left light darkish‐gray ringed tube is indole‐negative (done in the Haramaya University College of Veterinary Medicine Microbiological Laboratory).

**FIGURE 4:**
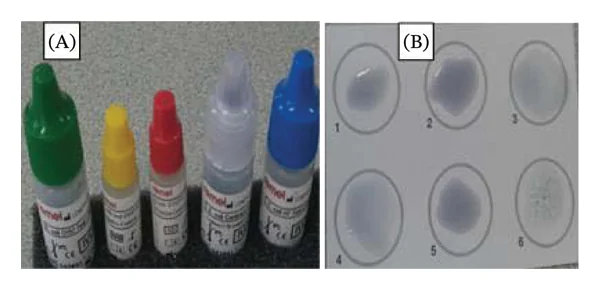
Latex test kit and latex agglutination test for *E. coli* O157:H7 in the laboratory: (A) Latex kit for *E.coli* O157:H7 test; (B) Latex agglutination test as the arrow showed positive result done in the Haramaya University College of Veterinary Medicine Microbiological Laboratory.

Upon multivariable regression analysis, the risk factors showed different levels of association to the occurrence of the targeted organisms as presented (Tables 4–6). As a result, immature fish had 2.1 times (95% CI = 1.0–4.5) the odds of being positive to E. coli O157:H7, and the association was significant (*P*=0.042), and “the female fish is 1.8 times (95% CI = 0.9–3.6) the odds of being positive to E. coli O157:H7, and the association was significant (*p* = 0.113)” as indicated (Table 4).

**TABLE 4:** Multivariable logistic regression analysis of risk factors for the occurrence of *E. coli* O157:H7 isolated from freshwater fish of Lake Abaya and Gilgel Gibe‐3 Dam.

Variables	Variable categories	No. of fish examined	No. (%) of fish positive to *E. coli* O157:H7	Adjusted odds ratio (95% CI)	*p* value
Sex	Male	147	16 (10.9)	^∗^	0.113
	Female	109	20 (18.3)	1.8 (0.9–3.6)	

Age	Mature	181	20 (11)	^∗^	0.042^∗^
	Immature	75	16 (21.3)	2.1 (1.0–4.5)	

*Note:* ^∗^The reference category/group used in the statistical analysis.

**TABLE 5:** Multivariable logistic regression analysis of risk factors for the occurrence of *E. tarda* isolated from freshwater fish of Lake Abaya and Gilgel Gibe‐3 Dam.

Variables	Variable categories	No. of fish examined	No. (%) of fish positive to *E. tarda*	Adjusted odds ratio (95% CI)	*p* value
Sex	Male	147	10 (6.8)	^∗^	0.248
	Female	109	12 (11)	1.7 (0.7–4.3)	

Age	Mature	181	11 (6.1)	^∗^	0.045^∗^
	Immature	75	11 (14.7)	2.6 (1.0–6.5)	

Lake	Abaya	170	10 (5.9)	^∗^	0.025
	Gilgel Gibe‐3 Dam	86	12 (14)	6.7 (1.3–35.7)	

Fish type	*O. niloticus*	68	8 (11.8)	3.7 (0.48–28.5)	0.207
	*C. gariepinus*	54	1 (1.9)	^∗^	
	*L. niloticus*	101	7 (6.9)	1.2 (0.3–4.3)	0.833
	*V. beso*	33	6 (18.2)	5.6 (0.6–52.6)	0.133

*Note:* ^∗^The reference category/group used in the statistical analysis.

**TABLE 6:** Multivariable logistic regression analysis of risk factors for the occurrence of *E*. *ictaluri* isolated from freshwater fish of Lake Abaya and Gilgel Gibe‐3 Dam.

Variables	Variable categories	No. of fish examined	No.(%) of fish positive to *E. ictaluri*	Adjusted odds ratio (95% CI)	*p* value
Sex	Male	147	9 (6.1)	3.2 (0.7–15.6)	0.146
	Female	109	3 (2.8)	^∗^	

Age	Mature	181	5 (2.8)	^∗^	0.023^∗^
	Immature	75	7 (9.3)	5.4 (1.3–23.3)	

Lake	Abaya	170	6 (3.5)	^∗^	0.246
	Gilgel Gibe‐3 Dam	86	6 (7.0)	2.3 (0.6–9.6)	

Fish type	*O. niloticus*	68	0	2.8	1.00
	*C. gariepinus*	54	12 (22.2)	1052166068.834	0.998
	*L. niloticus*	101	0	2.6	1.00
	*V. beso*	33	0	^∗^	

*Note:* ^∗^The reference category/group used in the statistical analysis.

Age of fish and lake showed significant association (*p* < 0.05) with the occurrence of E. tarda. Thus, immature fish and fishes from Gilgel‐Gibe had 2.6 (95% CI=1.0–6.5) and 6.7 (95% CI = 1.3–35.7) times the odds of being positive to E. tarda than their counter categories. Among the fish species, V. beso had 5.6 (95% CI = 0.6–52.6) the odds of being infected with E. tarda compared to *C. gariepinus* (Table 5).

The multivariable logistic regression analysis showed the relation of *E. ictaluri* to the assessed risk factors. Thus, the maturity of fish was found to be statistically significantly associated (*p* = 0.023) with the presence of the pathogen, such that the immature fish had 5.4 times (OR = 5.4; 95% CI = 1.3–23.3) the chance of having the pathogen as compared to the mature one (Table 6).

### 3.3. Prevalence of Isolated Bacteria Based on Sample Source

Out of the total of 768 fish tissues (i.e., intestine, liver, and kidney), 41 (5.34%), 31 (4.01%), and 15 (1.95%) were positive for *E. coli* O157:H7, *E. tarda*, and *E. ictaluri*, respectively. The majority of the *E. coli* O157:H7 (10.2%), *E. tarda* (4.7%), and *E. ictaluri* (2.3%) isolates were detected from the intestine. Meanwhile, a large proportion of *E. tarda* (4.3%) was isolated from the kidney. Overall, the intestine was found to harbor more frequent isolates (17.2%) than liver (8.98%) and kidney (7.9%), and there were statistically significant differences in the distribution of the isolates among different sample sources (*p* < 0.05) (Table 7).

**TABLE 7:** The prevalence of E. tarda, E. ictaluri, and E. coli O157:H7 from the sampled organ of fish.

Parameters		No. of fish examined	Prevalence (%)			Total
			*E. tarda*	*E. ictaluri*	*E. coli* O157:H7	
Sample source	Intestine	256	12 (4.7)	6 (2.3)	26 (10.2)	44 (17.2)
	Liver	256	8 (3.1)	5 (1.9)	10 (3.9)	23 (8.98)
	Kidney	256	11 (4.3)	4 (1.5)	5 (1.9)	20 (7.8)

	Total	768	31 (4.04)	15 (1.95)	41 (5.34)	87 (11.3)

*X* ^2^			87.386	82.264	61.595	

*p* value			0.000^∗^	0.001^∗^	0.000^∗^	

*Note:* ^∗^The variable/category showed statistically significant association.

### 3.4. Susceptibility to Antimicrobial Agents

Antimicrobial susceptibility test results (Table 8) showed that 100% susceptibility was noted for gentamycin, sulfamethoxazole, and trimethoprim by *E. tarda*, *E. ictaluri*, and *E. coli* O157:H7 isolates. Meanwhile, a large proportion of *E. tarda* (71%), *E. coli* O157:H7 (69%), and *E. ictaluri* (54%) were resistant to tetracycline.

**TABLE 8:** Antimicrobial response to bacterial isolates.

Drugs	*E. tarda* (*n* = 31)						*E. ictaluri* (*n* = 15)						*E. coli* O157:H7 (*n* = 41)					
	*S*		*I*		*R*		*S*		*I*		*R*		*S*		*I*		*R*	
	*N*	%	*N*	%	*N*	%	*N*	%	*N*	%	*N*	%	*N*	%	*N*	%	*N*	%
ST	22	71	5	16	4	13	11	73	3	20	1	6.7	28	68	10	24	3	7.3
CXT	23	74	8	26	0	0	13	87	2	13	0	0	21	51	11	27	9	22
AMP	24	77	7	23	0	0	13	87	2	13	0	0	28	68	5	12	7	17
TE	5	16	4	13	22	71	2	13	5	33	8	54	5	12	8	19	28	69
GEN	31	100	0	0	0	0	15	100	0	0	0	0	41	100	0	0	0	0
SUF	31	100	0	0	0	0	15	100	0	0	0	0	41	100	0	0	0	0
TRI	31	100	0	0	0	0	15	100	0	0	0	0	41	100	0	0	0	0

*Note:* ST, streptomycin; CXT, cefoxitin; AMP, ampicillin; TE, tetracycline; GEN, gentamycin; SUF, sulfamethoxazole; TRI, trimethoprim; S, susceptible; I, intermediate; R, resistant.

From the total isolates tested, only 9.7% of *E. tarda*, 6.7% of *E. ictaluri*, and 34.1% of *E. coli* O157:H7 isolates exhibited resistance against combinations of 2 antibiotics. A most frequent pattern consisting of two drugs was exhibited for cefoxitin and tetracycline as well as ampicillin and tetracycline by 14.6% of *E. coli* O15:H7 isolates (Table 9). Moreover, the majority of the isolates showed resistance to tetracycline.

**TABLE 9:** Multidrug resistance profiles of bacterial species isolated from fish.

Number	Resistant to:	Number of resistant isolates (%) in:		
		*E. tarda* (*n* = 31)	*E. ictaluri* (*n* = 5)	*E. coli* O15:H7 (*n* = 41)
One drug	TE	19 (61.3)	7 (46.7)	14 (34.1)
	ST	1 (3.2)	—	1 (2.4)
	FOX	—	—	2 (4.9)

Two drugs	ST, TE	3 (9.7)	1 (6.7)	2 (4.9)
	FOX, TE	—	—	6 (14.6)
	AMP, TE	—	—	6 (14.6)
	None	8 (25.8)	7 (46.7)	10 (24.4)

Total		31 (100)	15 (100)	41 (100)

*Note:* AMP, ampicillin; TE, tetracycline; FOX, cefoxitin; ST, streptomycin.

## 4. Discussion

### 4.1. Overall Prevalence of Bacterial Species in Fishes

In this study, a total of 256 fishes from Lake Abaya and Gilgel Gibe‐3 Dam were examined for the presence of bacteria, and 14.1%, 8.6%, and 4.7% were positive for *E. coli* O157:H7, *E. tarda*, and *E. ictaluri,* respectively. These findings are in agreement with the reports of Ref. [18] who reported 9.7% prevalence of *E. tarda* from Lake Langano and Ziway. Similarly, a study undertaken in Lake Ziway showed *E. coli* O157:H7 prevalence to be 12% [27]. In their study, Ref. [28] found lower prevalence of *E. coli* O157:H7 (2.4%), *E. tarda* (1.95%), and *E. ictaluri* (1.2%) from Lake Tana and slightly higher prevalence (19.05%) of *E. tarda* reported by Ref. [26] at Lake Ziway. The observed variation may be related to difference in sample size, sampling methods, environmental condition of the water (pH, temperature, salinity of the habitat, and bacterial load in the water) [46, 47], and proximity of Gilgel Gibe‐3 Dam to the Bele Town which lead to probability of water pollution. According to Ref. [48], the occurrence of an infection in a fish could not necessarily be an abnormal event; yet, it will lead to a disease situation. Moreover, under natural conditions, infectious agents coexist with their host without causing significant disease. However, stress factors are the main cause for the dissemination of infections and also for the occurrence of many disease outbreaks [48]. The presence of the bacteria in the internal organs of fish indicates the breakdown of immunological defense mechanisms [49].

### 4.2. Risk Factors for Prevalence of Bacterial Species in Fishes

*E. coli* O157:H7 and *E. tarda* were frequently isolated from female fish (18.3% and 11%), while *E. ictaluri* was the least frequently (2.8%) isolated bacteria from female fish, but there were no statistically significant differences. However, the maturity of fish showed statistically significant association with respect to *E. coli* O157:H7 (*p* = 0.031, *X* ^2^ = 4.6), *E. tarda* (*p* = 0.02, *X* ^2^ = 4.9), and *E. ictaluri* (*p* = 0.045, *X* ^2^ = 5.1) isolation, in that the prevalence was higher in immature fishes than mature ones.

The significant differences among maturation stages may be related to the change in the microenvironment of the intestine at different age levels and lack of immunological defense mechanisms in immature fish as well as related stress factors of the fish. These findings are in agreement with the report of Ref. [18, 47]. The absence of significant differences in the occurrence of isolated organisms between males and females indicates that both sexes are equally susceptible to the bacterium. This is in agreement with several works where both sexes have equal chance of being infected with bacteria [18, 28, 50].

The percentage of isolated bacteria was found to vary significantly among different fish species, study area, and organ of isolation. Thus, *E. tarda* was frequently isolated from *V. beso*, while *C. gariepinus* was the only fish to harbor *E. ictaluri*. With regard to the source of fish, the significant association (*p* < 0.05) was noted on *E. tarda* isolates, with higher occurrences in fishes from Gilgel Gibe‐3 Dam than Lake Abaya. Meanwhile, *E. ictaluri* occurrence was relatively higher (*p* = 0.226) in Gilgel Gibe (7.0%) than Lake Abaya (5.5%). The difference among fish species with respect to bacterial composition may be an immunological defense mechanism and variation in feeding habit among fish species [49].

In this study, *E. ictaluri* was detected only from catfish (*C. gariepinus*) with a prevalence of 22.2%. This is far higher than the finding of Ref. [28] in Lake Tana, who reported a prevalence of 1.2%. Although there is limited information about the disease occurrence in Ethiopia, *E*. *ictaluri,* it is a significant pathogenic bacterium known to cause enteric septicemia of catfish in fresh and marine fish species both cultured and wild fish [20].

Upon multivariable logistic regression analysis, the maturity of fish was found to be significantly associated (*p* = 0.045), in that immature fish had 2.6 times (OR = 2.6, 95% CI = 1–6.5) the chance of being infected by *E. tarda*. Moreover, fish from Gilgel Gibe‐3 Dam had 6.7 times the odds of harboring *E. tarda* than Lake Abaya, and the difference was statistically significant (*p* = 0.025). These findings are in agreement with Ref. [34]. This difference may be attributed to differences in the nutritional status of the fish, low level of salinity of water in Gilgel Gibe‐3 Dam, and moderate temperature (20°C–35°C) as reported by Ref. [30], which studied the water chemical characteristics and water pollution in Gibe‐3 Dam from Bele Town, pH, and fluctuation in dissolved oxygen. These factors contribute to variation in bacterial load due to the relative distance, and degree of exposure to the nearby source of pollution, which may also influence the frequency of bacterial isolation, and adequate temperature is also ideal for bacterial growth [48].

The characteristics of the microenvironment of the various sections of the alimentary tract of each fish species could also influence the taxonomic composition as well as the numerical abundance of the bacteria present [51]. The association of *E. coli* O157:H7 with sex was insignificant (*p* > 0.05). However, the maturity of fish was found to be significantly associated (*p* = 0.042) in that the presence of *E. coli* O157:H7 was 2.1 times (OR = 2.1, 95% CI = 1–4.5) more likely to be demonstrated in immature fish as compared to mature fish.

### 4.3. Prevalence of Bacterial Species Based on Tissues Examined

This study revealed that the overall prevalence of *E. tarda* in tissue samples (intestine, liver, and kidney) from apparently healthy fish was 4.01%. This finding is in agreement with Ref. [34], which indicate a prevalence of 3.2% from the Langano and Ziway lakes of Ethiopia. Ref. [52] reported respective prevalence of 3.44% in apparently healthy fish in Lake Hayike, Amhara Region, Northern Ethiopia. In contrast, Ref. [26, 34] in Lake Ziway and Hawasa found a high prevalence of *E. tarda* (20.59% and 19.05%, respectively). As compared to this study, Ref. [28], in Lake Tana, recorded a lower prevalence (1.9%). The difference in the reported prevalence could be associated with the sampling plan and procedure, environmental condition of the study area, sample type, bacteriological techniques employed in detecting *E. tarda,* or difference in occurrence and distribution of *E. tarda* in the study population regardless of test samples and methods of detection [28].

In this study, a 4.7% prevalence of *E. tarda* was recorded in intestinal samples. Previous reports by Ref. [53] (5.33%) in Lake Bishoftu and Ref. [34] (2.4%) in Lake Ziway from the intestine are consistent with the current findings. Around 3.1% *E. tarda* prevalence from the liver sample obtained in this study is in agreement with the prevalence (2%) report of Ref. [53] in Lake Bishoftu. Meanwhile, Ref. [34], in Lake Ziway, reported high prevalence (6.5%) from liver. The current finding of *E. tarda* prevalence from kidney (4.3%) is consistent with previous works in Lake Bishoftu (5.33%) by Ref. [28] but disagrees from that reported by Ref. [34] at a proportion of 0.8%. There was statistically significant (*p* < 0.05) variation in the prevalence of *E. tarda* among the three organs analyzed in this study, which is supported by the findings of Ref. [34] in the country. *E. ictaluri* from intestine, liver, and kidney had 2.3%, 1.9%, and 1.5% prevalence, respectively, with a significant difference (*p* < 0.05). The association of *E. ictaluri* with risk factors had no statistically significant association (*p* > 0.05) except maturity of fish (*p* = 0.023), and the OR was 5.4.

A 5.34% prevalence of *E. coli* O157:H7 was isolated in all types of samples, such as 10.2%, 3.9%, and 1.95% from intestine, liver, and kidney samples, respectively. These findings are in agreement with the reports of Ref. [54] who reported 2.3% from Lake Hawasa [27], 4% prevalence from intestine, liver, and kidney in Lake Ziway, and [28] 2.4% prevalence from Lake Tana. Moreover, the overall prevalence rate reported from different tissue samples (intestine, liver, and kidney) in our study is consistent with reports from other parts of the world, such as 7.56% from Nigeria [55] and 4.83% from Zimbabwe [56]. The samples taken from different fish species, namely Nile perch, Nile tilapia, catfish, and Belchi fish, for isolation and identification of *E. coli* O157:H7 are statistically significant (*p* < 0.05). This is in agreement with a previous report by Ref. [28] in Lake Tana.

### 4.4. Antimicrobial Susceptibility Pattern of Bacterial Isolates

The emergence and dissemination of antimicrobial resistance is on the increase among enteric bacteria [57]. Bacterial resistance to third‐generation cephalosporin is developed through the modification of enzymes called extended‐spectrum beta‐lactamase, which alter the activity of beta‐lactam antibacterial drugs. Extended‐spectrum beta‐lactamase is shared among bacteria and bacterial species [58]. The World Organization for Animal Health suggested that aquatic animal health should rely on constant monitoring and disease surveillance of antimicrobial‐resistant microbes that have interactions with aquatic animals [59].

All the *E. tarda, E. ictaluri*, and *E. coli* O157:H7 isolated in this study exhibited resistance to two or more antibiotics used in the study. Specifically, the isolates were 100% sensitive to gentamycin, sulfamethoxazole, and trimethoprim. All *E. tarda* and *E. ictaluri* were resistant to tetracycline and streptomycin, whereas *E. coli* O157:H7 resistance to tetracycline and cefoxitin were the most common resistance profiles identified among our study isolates. In the Ethiopian situation, limited studies report the antimicrobial susceptibility of *E. coli* O157:H7 isolated from apparently healthy fish. Thus, it was reported that the organism is highly sensitive to gentamycin, trimethoprim, sulfamethoxazole, and ciprofloxacin [54], while a high level of resistance was reported to cefoxitin, tetracycline, ampicillin, and streptomycin [54, 60]. The development of increasing multidrug‐resistant bacteria is signaling a serious alarm from a treatment point of view or the possible transfer of resistance genes to other related pathogens [61]. In this study, multiple antimicrobial resistances are also noted among *E. tarda, E. ictaluri*, and *E. coli* O157:H7 isolates drawn from different sample types. From the total tested *E. coli* O157:H7, *E. tarda*, and *E. ictaluri*, 34.1%, 9.7%, and 6.7% were resistant against combinations of two antibiotics, respectively, which was in consistent with the reports of Ref. [61]. They found that out of the 29 *E. coli* O157:H7 tested, four isolates indicated multiple resistances to five antimicrobials: tetracycline, ampicillin, streptomycin, kanamycin, and sulfamethoxazole. Two isolates originated from cattle, and two isolates were from humans and ground beef. It is important to keep up with isolate characteristics for any global changes in isolate distribution and similarities and prevalence of common virulence factors. It is also essential to track the tolerance pattern recorded globally to follow changes in antimicrobial sensitivity patterns that may need an assessment of control strategy. Monitoring antimicrobial resistance in *E. tarda, E. ictaluri*, and *E. coli* O157:H7 isolates is essential for epidemiological purposes and for tracking the rise in antimicrobial resistance across different bacterial species [61].

## 5. Conclusion

In this study, *E. tarda, E. ictaluri*, and *E. coli* O157:H7 were found to be common bacteria isolated from apparently healthy fish harvested from Lake Abaya and Gilgel Gibe‐3 Dam. The isolated bacteria were found to vary significantly among different fish species, study area, maturation stage, and organ of isolation. The observed difference may be due to sample size, sampling procedure, and bacteriological method employed, as well as factors, such as climatic condition, nutritional status of fish species in the lakes, and water pollution contributing to variation in bacterial load. The study also revealed a significant proportion of resistant isolates to the tested antibiotics, with a relatively high tolerance to tetracycline while exhibiting 100% sensitivity to gentamicin, trimethoprim, and sulfamethoxazole. The indiscriminate use of antibiotics to treat bacterial infections in both animals and humans can lead to the emergence of drug‐resistant bacteria. To address this issue, a multidisciplinary approach is necessary, including an understanding of the characteristics of pathogenic bacteria affecting fish, a better grasp of fish biology, and consideration of environmental factors to ensure effective fish disease control. Therefore, this study creates momentum for more research work to establish the occurrence and prevalence of *E. tarda, E. ictaluri*, and *E. coli* O157:H7 in lakes and dams and to determine cost‐effective measures that should be taken to reduce fish mortality.

## Author Contributions

Belay Walelign Moges led the investigation and project administration, coordinated the research activities, was responsible for the data collection and analysis, and prepared the original draft of the manuscript.

Jelalu Kemal Birmeka contributed to the design of the study, supervised the research activities, provided critical revisions to the manuscript, and contributed to the interpretation of the results.

Bruk Abraha Fitwi contributed to the conceptualization and design of the study, supervised the research activities, provided critical revisions to the manuscript, and contributed to the interpretation of the results.

Bulako Chebo Boke contributed for the collection of samples, conducted the laboratory experiments and analysis, managed data curation, and contributed to the initial drafting of the manuscript.

Tesfahun Demeke Dana contributed to data validation and editing and revision of the original draft of the manuscript.

Tesfaye Bekele Kassa played a significant role in the development of the methodology, provided technical guidance throughout the research, performed data analysis, participated in the review and editing of the manuscript, and served as the corresponding author.

## Funding

No funding was received for this research.

## Disclosure

The manuscript was previously presented as thesis [63] in Haramaya University, Ethiopia.

## Conflicts of Interest

The authors declare no conflicts of interest.

## Data Availability

The data that support the findings of this study are available upon request from the corresponding author.
